# Development of highly-multiplexed SNP arrays in maritime pine for multi-objective genetic applications

**DOI:** 10.1186/1753-6561-5-S7-P36

**Published:** 2011-09-13

**Authors:** C Plomion, E Chancerel, C Lepoittevin, I Lesur, SC González-Martínez, GG Vendramin, MT Cervera

**Affiliations:** 1 INRA, UMR1202 BIOGECO, France; 2 INIA, Center of Forest Research (CIFOR), Indonesia; 3 Italian National Research Council, Institute of Plant Genetics, Italy

## 

Single Nucleotide Polymorphisms (SNPs) are the most abundant form of genetic variation in the genome. In this poster, we reviewed what has been achieved in terms of highly-multiplexed SNP genotyping assay construction in maritime pine (*Pinus pinaster* Ait.), the main conifer used for commercial plantation in southwestern Europe. Seven custom SNP-assays (384 and 1536-plex), oriented towards broad applications, have been designed. We illustrated here the usefulness of this genotyping technology to address specific questions related to i/ genetic diversity and population structure analysis, ii/ linkage and QTL mapping and iii/ association mapping.

With respect to genetic diversity and differentiation, a custom VeraCode assay for 384 SNPs mostly based on a larger array designed for linkage mapping (see below) allowed to obtain less blurred, albeit similar, breeding zone boundaries than a set of 12 nuSSRs screened on the same individuals. Levels of diversity were also more accurately estimated, showing clear differences among gene pools. Interestingly, a relatively small subset of SNPs would be enough to develop an application tool for origin certification, which could have a notable impact on current operational practices.

In terms of linkage mapping, a custom GoldenGate assay for 1,536 SNPs detected through the resequencing of gene fragments (in vitro SNPs) and from Sanger-derived Expressed Sequenced Tags (in silico SNPs) was established. Offspring from two mapping pedigrees were genotyped. A consensus map comprising 357 SNPs from 292 different loci was constructed and the analysis of sequence homology between mapped markers and their orthologs in a *Pinus taeda* linkage map, made it possible to align the 12 linkage groups of both species. Moreover, QTL detection for different traits is underway.

In terms of association mapping, a custom GoldenGate assay with 384 SNPs was built and used to genotype 160 unrelated plus-trees from a half-sib experimental design for which breeding values (for height growth, circumference, stem straightness at 8 years, lignin content and extractives at 31 years) were available. Taking into account multiple testing, one single SNP in a gene encoding a putative fasciclin-like arabinogalactan protein was found to be associated with growth traits.

We conclude that the VeraCode/GoldenGate assays can be used successfully for high-throughput SNP genotyping in maritime pine, a conifer species that has a genome seven times the size of the human genome. This first generation of SNP-arrays has been recently upgraded to an Infinium-array (containing 10.5k SNPs) thanks to deep sequencing based on new generation sequencing technologies. The Infinium-array also includes SNPs from comparative orthologous sequences with other major conifer species, providing a wider collection of anchor points for comparative genomics among these major groups of forest trees.

